# Urinary bladder stone due to retained indwelling ureteral stent: A case report

**DOI:** 10.1097/MD.0000000000022293

**Published:** 2020-09-25

**Authors:** Fuxun Zhang, Jianhong Yu, Qianlong Wang, Yiping Lu

**Affiliations:** a Department of Urology, Institute of Urology, West China Hospital, Sichuan University, Chengdu, Sichuan; b Department of Urology, the Affiliated Hospital of Gansu Medical College, Pingliang, Gansu, China.

**Keywords:** bladder stone, calculi, complications, lithotripsy, ureteral stent

## Abstract

**Rationale:**

The indwelling ureteral stents is a common procedure in routine urological practice. The double-J (D-J) stent is the most common type of stents used and is indicated mainly for short-term urinary drainage and prevention of obstruction and infection. However, prolonged indwelling stents may result in disastrous complications, such as hematuria, infection, encrustation, and stone formation. In this context, the persistence of stent in situ might play a key role as a nidus in deposition of urinary sediment, then forming calculus. Although the encrustation may become more serious as time goes on, large bladder stones are relatively rare. However, the serious encrustation and giant stone may complicate or exacerbate the conditions in turn.

**Patient concerns:**

A 45-year-old female patient who underwent right ureteral stent placement after open ureterolithotomy 6 years ago complained of dysuria, urinary frequency, and urgency over 2 months.

**Diagnosis:**

The kidney ureter bladder (KUB) x-ray showed the presence of a giant stone in the bladder and an entire D-J stent. The computed tomography (CT) urography scans revealed normal left kidney, right hydronephrosis, and an encrusted D-J stent with the significant stone, diameter 4.2 cm with a CT value of 1211.0 ± 221.6 HU, on the vesical coil. On the basis of these auxiliary examinations, the case was diagnosed as cystolith and prolonged-indwelling stents.

**Interventions:**

Pneumatic ballistic lithotripsy was used for crushing the bladder calculi followed by the successful extraction of intact D-J ureteral stent.

**Outcomes:**

No residual stone was detected on postoperative KUB x-ray and CT urography scans. Patient recovered well and was discharged 10 days after surgery. Semi-annual ultrasound examination was suggested to monitor the effect of therapy.

**Lessons:**

This case reminds us that it is crucial to take various measures to avoid the forgotten ureteral stent and its unfortunate late complication.

## 1 Introduction

Ureteral stents are frequently used in the management of nephroureterolithiasis, genitourinary trauma, genitourinary reconstruction, and ureteral obstruction induced by malignancy or retroperitoneal fibrosis.^[1]^ The indications for ureteral stents, particularly double-J (D-J) stents, have been extended with improvement of stent materials and design in recent years. However, serious complications of stents placement are more common than in the past, such as hematuria, infection, fragmentation, encrustation, and stone formation. Especially, the serious encrustation and giant stone formation may complicate the condition and lead to renal function impairment in turn.^[2,3]^ Although the maximal time keeping a stent in the ureter without severe complications is not well defined, indwelling ureteral stents longer than initially planned even leaving it forgotten to remove following insertion is unsafe, sometimes life-threatening.^[4,5]^ The approaches, open or endourological surgery, for treatment of forgotten stents are ambiguous and considered difficult for patients with stent-related complications.^[5]^ Herein, we report a rare case of giant bladder stone caused by retained D-J stent which was not removed for 6 years and its endourological management utilizing conventional endourological procedures.

## 2 Case report

A 45-year-old woman was admitted to hospital with complaints of dysuria, urinary frequency, and urgency over 2 months. This patient had undergone right ureteral stent placement after successful open ureterolithotomy for an obstructing 1.0 cm ureteral calculi 6 years prior to presentation. A follow-up appointment had been scheduled, but the patient did not attend and was lost to follow-up subsequently.

**Figure 1:**
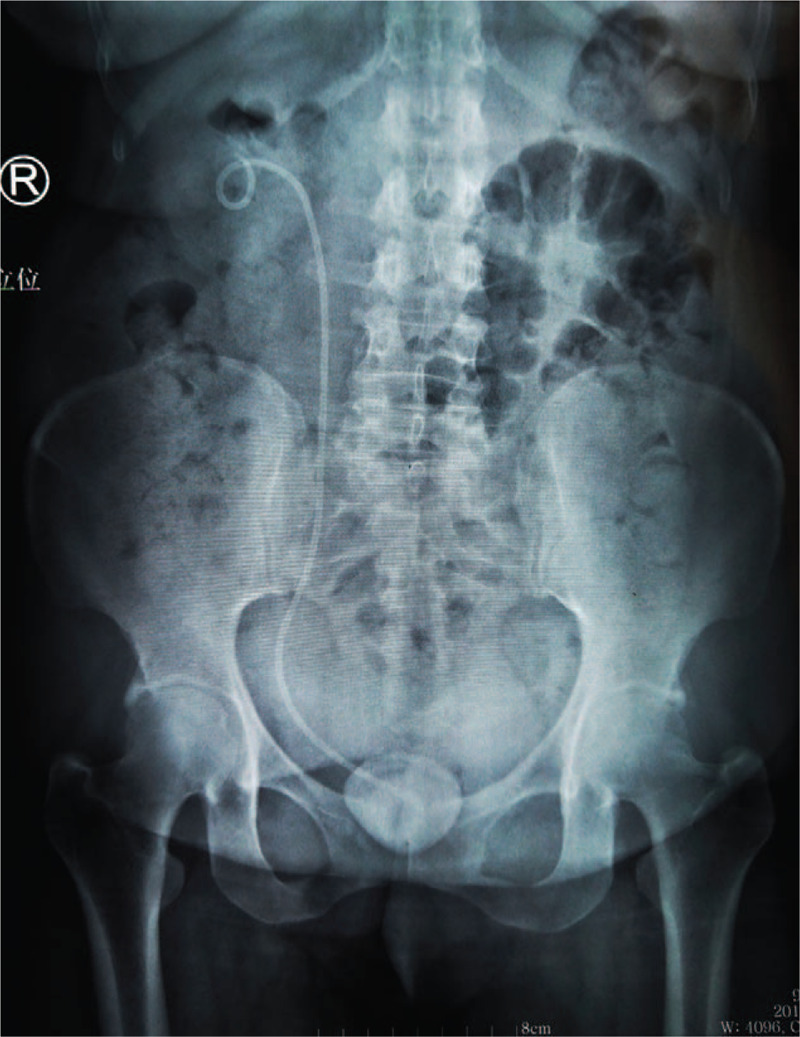
Preoperative KUB x-ray showed an entire coiled D-J stent with a giant stone in the bladder. D-J stent = double-J stent, KUB = kidney ureter bladder.

**Figure 2:**
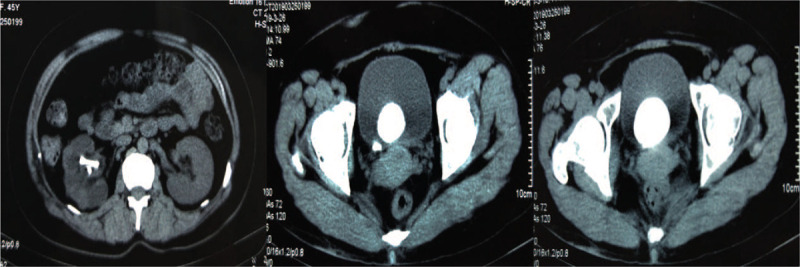
Giant bladder stone, an encrusted D-J stent, normal left kidney, and right hydronephrosis on CT scans. CT = computed tomography, D-J stent = double-J stent.

Physical examination was unremarkable at admission except mild tenderness above the symphysis pubis and a previous surgical scar. Her hematologic and biochemical investigations were also unremarkable. Urinalysis revealed microhematuria and pyuria (125 white blood cells and 80 red blood cells/high-powered field). Kidney ureter bladder (KUB) x-ray showed the presence of an entire coiled D-J stent with a giant stone in the bladder at the distal stent (Fig. 1). The computed tomography (CT) urography scans revealed normal left kidney, right hydronephrosis, and an encrusted D-J stent with the significant stone, diameter 4.2 cm with a CT value of 1211.0 ± 221.6 HU, on the vesical coil (Fig. 2).

**Figure 3:**
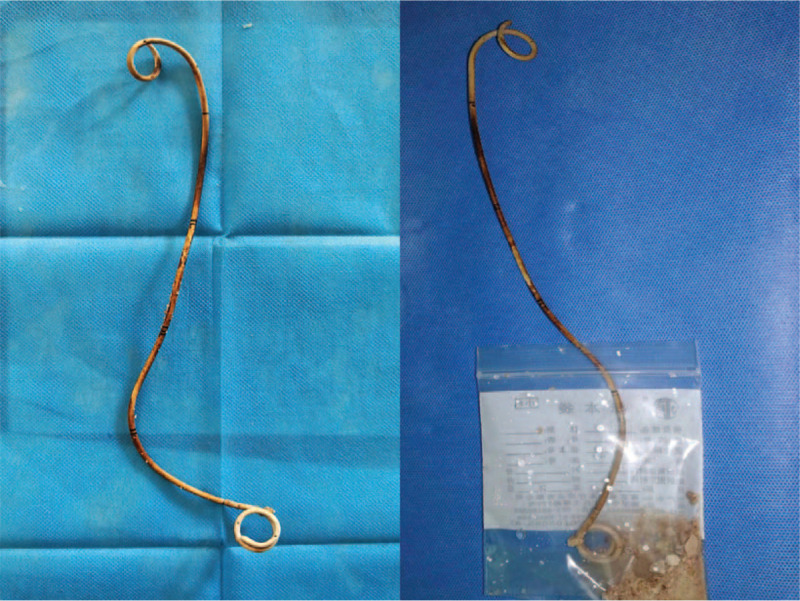
Intact D-J ureteral stent with uncomplicated stent encrustation following removal. D-J stent = double-J stent.

**Figure 4:**
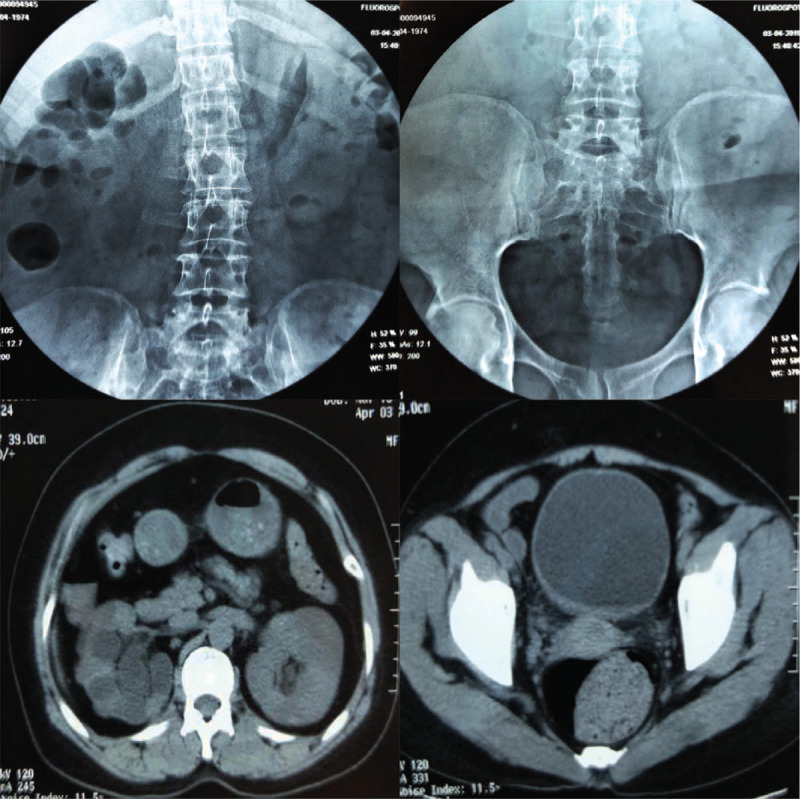
No residual kidney and bladder stone was detected on postoperative KUB x-ray and CT scans. CT = computed tomography, KUB = kidney ureter bladder.

Following treatment for the urinary tract infection, the bladder stone was crushed with pneumatic ballistic lithotripsy via nephroscope under spinal anesthesia and the intact D-J ureteral stent was withdrawn using a grasper successfully (Fig. 3). The whole procedure for lithotripsy combined D-J stent removal took 90 minutes. The urethral catheter was removed 3 days later and no residual bladder stone was detected on postoperative KUB x-ray and CT urography scans (Fig. 4). Patient, recovered well and no complications occurred, was discharged 10 days after surgery. Semi-annual ultrasound examination was recommended to monitor calculus recurrence and progression of right hydronephrosis. The Institutional Ethics Committee of the West China Hospital had approved this case report and the patient had provided written consent for publication of the case.

## 3 Discussion

The D-J ureteral stent is being frequently used as an almost routine procedure following endourological or urological open surgery in cases of ureteral obstructive conditions. Placement of a D-J stent following nephroureterolithotomy is intended to prevent obstruction of the urinary tract and deterioration of renal function, and it is suggested that ureteral stents should be inserted in patients who are under the risk of complications.^[6]^ However, complications associated to indwelling ureteral stents are often encountered and could result in significant morbidity.^[7]^ Side effects and complications divided by timing include early complications such as iatrogenic injury in the process of placement, irritative bladder symptoms, hematuria or bacteriuria, and delayed complications such as stent migration, fragmentation, encrustation, or stone formation.^[7,8]^ A major risk factor for stent encrustation is long-term placement of stents in patients with poor compliance to follow-up, also known as forgotten stents.

Although the optimum interval for removing an indwelling ureteral stent is ambiguous, long-term placement of ureteral stents might give rise to increase of risk for stent encrustation or stone formation.^[9]^ In our case, giant bladder calculus formed after the D-J stent was inserted 6 years ago. The stone formation was being developed from the vesical distal stent, namely nidus, to versical coil and the stone accretion was inevitable. Surprisingly, due to severe stent encrustation which is difficult to handle and always impedes the process of stent removal did not occur in this case, the D-J stent was extracted smoothly after cystolithotripsy without further ureterolithotripsy. In view of ipsilateral hydronephrosis, we hold the opinion that insufficiency and urinary-hyposecretion of involved kidney might be responsible for the uncomplicated stent encrustation.

In conclusion, the forgotten stent profiles an unfortunate late complication of indwelling stent, because it is caused by human factors and thus totally preventable. With extension of stent-dwelling interval, risk of encrustation or stone formation and difficulty of stent extraction will arise considerably, sometimes deadly.^[10]^ Various measures should be implemented to avoid forgotten stents, including computerized stent-tracking registry, automatic appointment reminders, and bar coded wrist bands. Meanwhile, it is essential to elucidate the complications of long-term indwelling stents in detail for reducing non-compliance of the patients. In spite of these methods to avoid this urological dilemma, urologists should have a more important role to play in averting these unnecessary operation-related complications.

## Acknowledgments

All named authors meet the International Committee of Medical Journal Editors (ICMJE) criteria for authorship for this article, take responsibility for the integrity of the work as a whole, and have given their approval for this version to be published. The authors thank the participants of the study.

Support: 1.3.5 project for disciplines of excellence. West China Hospital, Sichuan University.

## Author contributions

**Conceptualization:** Fuxun Zhang.

**Data curation:** Fuxun Zhang.

**Formal analysis:** Fuxun Zhang.

**Funding acquisition:** Fuxun Zhang.

**Investigation:** Fuxun Zhang, Jianhong Yu, Qianlong Wang.

**Methodology:** Fuxun Zhang.

**Project administration:** Fuxun Zhang.

**Resources:** Fuxun Zhang, Jianhong Yu, Qianlong Wang.

**Software:** Fuxun Zhang.

**Supervision:** Fuxun Zhang.

**Validation:** Fuxun Zhang, Jianhong Yu, Qianlong Wang.

**Visualization:** Fuxun Zhang.

**Writing – original draft:** Fuxun Zhang.

**Writing – review & editing:** Fuxun Zhang, Yiping Lu.
